# Evaluating the impact of alternative phenotype definitions on incidence rates across a global data network

**DOI:** 10.1093/jamiaopen/ooad096

**Published:** 2023-11-21

**Authors:** Rupa Makadia, Azza Shoaibi, Gowtham A Rao, Anna Ostropolets, Peter R Rijnbeek, Erica A Voss, Talita Duarte-Salles, Juan Manuel Ramírez-Anguita, Miguel A Mayer, Filip Maljković, Spiros Denaxas, Fredrik Nyberg, Vaclav Papez, Anthony G Sena, Thamir M Alshammari, Lana Y H Lai, Kevin Haynes, Marc A Suchard, George Hripcsak, Patrick B Ryan

**Affiliations:** OHDSI Collaborators, Observational Health Data Sciences and Informatics (OHDSI), New York, NY 10027, United States; Global Epidemiology, Janssen Pharmaceutical Research and Development, LLC, Titusville, NJ 08560, United States; OHDSI Collaborators, Observational Health Data Sciences and Informatics (OHDSI), New York, NY 10027, United States; Global Epidemiology, Janssen Pharmaceutical Research and Development, LLC, Titusville, NJ 08560, United States; OHDSI Collaborators, Observational Health Data Sciences and Informatics (OHDSI), New York, NY 10027, United States; Global Epidemiology, Janssen Pharmaceutical Research and Development, LLC, Titusville, NJ 08560, United States; OHDSI Collaborators, Observational Health Data Sciences and Informatics (OHDSI), New York, NY 10027, United States; Department of Biomedical Informatics, Columbia University Irving Medical Center, New York, NY 10027, United States; OHDSI Collaborators, Observational Health Data Sciences and Informatics (OHDSI), New York, NY 10027, United States; Department of Medical Informatics, Erasmus University Medical Center, Rotterdam, 3000 CA, The Netherlands; OHDSI Collaborators, Observational Health Data Sciences and Informatics (OHDSI), New York, NY 10027, United States; Global Epidemiology, Janssen Pharmaceutical Research and Development, LLC, Titusville, NJ 08560, United States; OHDSI Collaborators, Observational Health Data Sciences and Informatics (OHDSI), New York, NY 10027, United States; Fundació Institut Universitari per a la recerca a l'Atenció Primària de Salut Jordi Gol i Gurina (IDIAPJGol), Barcelona, 08007, Spain; Research Programme on Biomedical Informatics (GRIB), Hospital del Mar Medical Research Institute (IMIM), Barcelona, 08003, Spain; Management Control Department, Parc de Salut Mar (PSMAR), Barcelona, 08007, Spain; Research and Development, Heliant d.o.o, Belgrade, 11000, Serbia; Institute of Health Informatics, University College London, London, NW1 2DA, United Kingdom; British Heart Foundation Data Science Centre, HDR, London, NW1 2DA, United Kingdom; School of Public Health and Community Medicine, Institute of Medicine, Sahlgrenska Academy, University of Gothenburg, Gothenburg, 40530, Sweden; Institute of Health Informatics, University College London, London, NW1 2DA, United Kingdom; OHDSI Collaborators, Observational Health Data Sciences and Informatics (OHDSI), New York, NY 10027, United States; Global Epidemiology, Janssen Pharmaceutical Research and Development, LLC, Titusville, NJ 08560, United States; Department of Medical Informatics, Erasmus University Medical Center, Rotterdam, 3000 CA, The Netherlands; OHDSI Collaborators, Observational Health Data Sciences and Informatics (OHDSI), New York, NY 10027, United States; College of Pharmacy, Prince Sattam Bin Abdulaziz University, Riyadh, 11942, Saudi Arabia; OHDSI Collaborators, Observational Health Data Sciences and Informatics (OHDSI), New York, NY 10027, United States; Division of Informatics, Imaging and Data Sciences, University of Manchester, Manchester, M13 9PL, United Kingdom; Global Epidemiology, Janssen Pharmaceutical Research and Development, LLC, Titusville, NJ 08560, United States; OHDSI Collaborators, Observational Health Data Sciences and Informatics (OHDSI), New York, NY 10027, United States; Department of Biostatistics, University of California, Los Angeles, Los Angeles, CA 90025, United States; OHDSI Collaborators, Observational Health Data Sciences and Informatics (OHDSI), New York, NY 10027, United States; Department of Biomedical Informatics, Columbia University Irving Medical Center, New York, NY 10027, United States; OHDSI Collaborators, Observational Health Data Sciences and Informatics (OHDSI), New York, NY 10027, United States; Global Epidemiology, Janssen Pharmaceutical Research and Development, LLC, Titusville, NJ 08560, United States; Department of Biomedical Informatics, Columbia University Irving Medical Center, New York, NY 10027, United States

**Keywords:** phenotype, electronic health record, algorithms, incidence study

## Abstract

**Objective:**

Developing accurate phenotype definitions is critical in obtaining reliable and reproducible background rates in safety research. This study aims to illustrate the differences in background incidence rates by comparing definitions for a given outcome.

**Materials and Methods:**

We used 16 data sources to systematically generate and evaluate outcomes for 13 adverse events and their overall background rates. We examined the effect of different modifications (inpatient setting, standardization of code set, and code set changes) to the computable phenotype on background incidence rates.

**Results:**

Rate ratios (RRs) of the incidence rates from each computable phenotype definition varied across outcomes, with inpatient restriction showing the highest variation from 1 to 11.93. Standardization of code set RRs ranges from 1 to 1.64, and code set changes range from 1 to 2.52.

**Discussion:**

The modification that has the highest impact is requiring inpatient place of service, leading to at least a 2-fold higher incidence rate in the base definition. Standardization showed almost no change when using source code variations. The strength of the effect in the inpatient restriction is highly dependent on the outcome. Changing definitions from broad to narrow showed the most variability by age/gender/database across phenotypes and less than a 2-fold increase in rate compared to the base definition.

**Conclusion:**

Characterization of outcomes across a network of databases yields insights into sensitivity and specificity trade-offs when definitions are altered. Outcomes should be thoroughly evaluated prior to use for background rates for their plausibility for use across a global network.

## Objective

The objective of this study is to evaluate the impact of phenotype modification (outcome definition) on the incidence rate of 13 adverse events of special interest (AESI) for coronavirus disease 2019 (COVID-19) vaccine monitoring, estimated from real-world data. The modifications include restricting to events that occur in an inpatient setting, change in the code set used to capture the events, and use of standardized vocabulary to derive the code set. The 13 AESIs for COVID-19 vaccine monitoring are outcomes that are considered important to monitor as known potential risks related to COVID-19 vaccines or vaccination in general.

## Background and significance

COVID-19 vaccines were authorized for emergency use in late 2020. Researchers and regulators have prepared safety surveillance approaches that involve real-world data to study AESIs of the vaccines. AESIs need to be monitored because not all possible adverse events are expected to occur during the pre-approval clinical studies; these vaccines were also approved under emergency use and approved in unstudied populations such as children and pregnant women.^1^^,^^2^ The United States Food and Drug Administration (US FDA), Centers for Disease Control and Prevention (CDC), and the vACCine covid-19 monitoring readinESS (ACCESS) project funded by the European Medicines Agency (EMA) have provided protocols to monitor the safety of COVID-19 vaccines.^3^^,^^4^ All such protocols have listed a number of AESIs and each provided a computable specific definition that can be implemented against real-world data to capture these events. As such, the AESIs represent a collection of outcomes or disease states that can be defined in data sources and then applied across data sources to represent the outcome to be used in safety surveillance studies for those receiving the vaccine.^5^

Background incidence rates play an integral part in vaccine safety surveillance, as these rates are commonly compared to incidence rates of adverse events following vaccination to determine whether adverse event reporting rates are higher than expected. Studies often report wide variability of these rates across data sources and populations. Li et al.^6^ found that incidence rates had a high level of population-level heterogeneity across databases after standardizing the definition and stratifying on age and sex. Ostropolets et al.^7^ examined the factors that influence variability in incidence rates, including demographics of the population, such as age and sex distributions, along with choices around time-at-risk and anchoring of rates (anchoring on healthcare provider visits or random dates). They concluded that population-level characteristics have the greatest influence on rates, and rates are highly influenced by time-at-risk start dates. These 2 studies document the variability in incidence rates related to population-level adjustment and parameters used to calculate the rates. The influence of phenotype choices on incidence rates remains unknown.

Computable phenotypes, or definitions of a disease in databases, have been studied widely in observational research. These definitions can be developed from literature, prior research or clinical information, or use of systematic vocabularies and ontologies.

Regulatory bodies such as US FDA or EMA commonly use country-specific vocabularies, including the International Classification of Diseases, Tenth Revision, Clinical Modification (ICD-10-CM) in the United States, to define clinical events. However, large-scale studies across a network using data from multiple countries and terminologies require the use of standardization. The Observational Health Data Sciences and Informatics (OHDSI) provides the Observational Medical Outcomes Partnership (OMOP) Common Data Model (CDM) with a set of vocabularies to harmonize and standardize source terminologies.

While regulatory bodies define computable phenotypes for these outcomes, the specific impact and relevance of these definitions to clinical populations remain unclear. The definitional logic is often under-reported in scientific journals, and definitions are often created utilizing various evaluation methods and designs without using global data.^2^^,^^8^ COVID-19 vaccines are being administered all over the world, and understanding how to identify adverse events in global data is critical for the safety of the patients that receive these vaccines. Understanding the distinctive differences among alternative definitions (sensitive and specific) and how best to implement them in each data source are imperative to correctly apply an outcome for safety monitoring. The impact of changing outcome definitions in a network of global databases remains unknown.

As provided in different regulatory protocols, outcome phenotypes mainly varied in 3 different ways: first, restricting events that only occurred in an inpatient setting or including events regardless of the setting of service; second, including a different set of event codes (eg, disease diagnosis codes) to capture an event of interest; and finally, the use of different vocabulary/ontology systems to code for clinical events.^3^ For example, Guillain-Barre syndrome (GBS) can be defined using inpatient restrictions or any place of service while definitions for hemorrhagic stroke could include a broad range of codes for lacunar infarctions or choosing not to include them.

In this study, we sought to assess the impact of 3 types of phenotype modification. While changes to phenotypes can be innumerable and extend beyond what is presented here (including baseline characteristics, code set changes, sites etc.) our focus is on selected factors that could influence baseline incidence rates. Specifically, we estimated the difference in incidence rates of outcomes using: (1) definitions that restricted to an inpatient setting compared to any care setting for the same event of interest; (2) definitions that included a different set of codes to capture the same event of interest; and (3) definitions that used country-specific (which we have termed “source”) vocabularies compared to using a common vocabulary that provides semantic standardization.^9^

## Materials and methods

We conducted an international network study using routinely collected primary care and hospital patient records from across the United States, Australia, Japan, and Europe. To be included in the study, each data source needed to have data for the specified study calendar time from January 1 to December 31 for each qualifying year in 2017-2019 to be included in the study, see Appendix Figure S1 for the study design of the entry criteria. Each data source mapped their data to the OMOP CDM.^10^^,^^11^ This approach allows contributing data sites to execute an analytical package in R to calculate background rates and descriptive characteristics in a federated fashion.^12^ The analytical code to characterize these phenotype definitions can be found here: https://github.com/ohdsi-studies/Covid19VaccineAesiDiagnostics.^13^ The package to estimate background rates can be found here: https://github.com/ohdsi-studies/Covid19VaccineAesiIncidenceRate.

### Data sources

We included 16 data sources from 10 countries, of which 5 data sources were claims and the remaining were electronic health records (EHRs).

The claims-based data sources were (1) JMDC (JMDC_JAPAN)^14^ and 4 US administrative claims data sources: (2) IBM MarketScan Commercial Claims and Encounters Database (CCAE_US); (3) IBM MarketScan Medicare Supplemental Database (MDCR_US); (4) IBM MarketScan Multi-State Medicaid Database (MDCD_US); and (5) Optum De-Identified Clinformatics Extended Data Mart Database—Date of death (OPTUM_DOD_US).

The EHR data sources were: (1) IQVIA Australia Longitudinal Patient Data (LPD) (IQVIA_AUSTRALIA), data collected from Australian general practitioner (GP) offices; (2) Integrated Primary Care Information (IPCI_NETHERLANDS), a primary care records data source from the Netherlands^15^; (3) IQVIA Disease Analyzer (DA) Germany (IQVIA_GERMANY), data collected from physician practices and medical centers; (4) Clinical Practice Research Datalink (CPRD), which consists of data collected from United Kingdom primary care for all ages (CPRD_UK); (5) Columbia University Irving Medical Center (CUMC_US), which covers the New York-Presbyterian Hospital/Columbia University Irving Medical Center in the United States; (6) Optum de-identified Electronic Health Record Dataset (OPTUM_EHR_US), which covers more than 103 million patients and over 7000 hospitals and clinics across the United States; (7) Health Data Warehouse of Assistance Publique—Hopitaux de Marseille in France (APHM_FRANCE), a public university hospital system with 4 hospitals, 3400 beds, and more than 12 000 healthcare professionals; (8) Information System of Parc Salut Mar Barcelona (PSMAR_SPAIN), hospital based EHR that includes 2 general hospitals and 2 clinics in Barcelona, Spain; (9) University Clinical Center of Serbia (CC_SERBIA), a hospital based EHR data from Serbia; (10) Health Informatics Centre from University of Dundee (HIC_SCOTLAND), a hospital based EHR dataset from Scotland; and (11) UK Biobank (BIOBANK_UK), a large longitudinal biobank study from the United Kingdom with linkages to primary care and hospitalization EHR.^16^

A detailed description of the data sources can be found in Appendix Table S1. The data underlying this article were provided by [third party] under license/by permission.

### Study population

The study population consisted of individuals present in a data source as of January 1, 2017, 2018, or 2019 and is defined as the index date. Individuals were required to have a minimum of 1 year of history available in the data source prior to the index date. A minimum of 1 year of history is defined as having at least 1 year observation time prior to index date. Observation start time is defined either through enrollment files or visit encounters depending on the data source.

### Outcomes

Tables 1-3 illustrates the full set of outcomes used in the study and the type of modification each represents. For each outcome, a base definition was developed. All base definitions were based on a specific code set of standard SNOMED-CT, each SNOMED-CT is mapped to source codes in various source codes such as ICD10-CM, Read codes etc., and these events at any place of service. Place of service varies from inpatient stays, emergency room, outpatient encounters, and a combined visit (IP/ER) which is used when emergency room and inpatient stays cannot be separated into individual encounters. For hemorrhagic and non-hemorrhagic strokes, the base definition restricted to inpatient setting as diagnosis and treatments occur in a hospitalized setting for new events. To assess the impact of restricting on a place of service, additional cohorts for inpatient-only were considered for the following outcomes: acute myocardial infarction (MI), anaphylaxis, appendicitis, deep vein thrombosis (DVT), disseminated intravascular coagulation (DIC), encephalomyelitis, GBS, and transverse myelitis as these events can occur any setting. For example, GBS is defined with the same codes and definition and is compared to a subset of GBS patients that had an encounter of inpatient.

**Table 1.** Outcomes evaluated by inpatient restriction.

Inpatient restriction				
Outcomes (number of comparisons)	Base definition (SNOMED-CT terms and its descendants unless otherwise noted)		Inpatient definition	
	Definition	Place of service	Definition	Place of service
Acute myocardial infarction (2)	Myocardial infarction, ventricular aneurysm due to and following acute myocardial infarction; thrombosis of atrium, auricular appendage, and ventricle due to and following acute myocardial infarction; pulmonary embolism due to and following acute myocardial infarction; mitral valve regurgitation due to and following acute myocardial infarction; hemopericardium due to and following acute myocardial infarction; cardiac rupture due to and following acute myocardial infarction; atrial septal defect due to and following acute myocardial infarction; arrhythmia due to and following acute myocardial infarction; excluding including descendants: old myocardial infarction	IP, ER, OP	Same as base definition	IP
Anaphylaxis (2)	Anaphylaxis, anaphylactic shock due to serum	IP, ER, OP, IP/ER	Same as base definition	IP, IP/ER
Appendicitis (2)	Appendicitis	IP, ER, OP, IP/ER	Same as base definition	IP, IP/ER
Deep vein thrombosis (1)	Embolism from thrombosis of vein of lower extremity; embolism and thrombosis of the vena cava; embolism and thrombosis of the renal vein; deep venous thrombosis; acute deep venous thrombosis of internal jugular vein; acute deep venous thrombosis of axillary vein; parent codes only: venous thrombosis; thrombosis of vein of lower limb; excluding including descendants: postpartum deep phlebothrombosis; deep vein phlebitis and thrombophlebitis of the leg; chronic deep venous thrombosis; axillary vein thrombosis; antepartum deep vein thrombosis	IP, ER, OP	Same as base definition	IP
Disseminated intravascular coagulation (1)	Disseminated intravascular coagulation	IP, ER, OP	Same as base definition	IP
Encephalomyelitis (2)	Encephalomyelitis, encephalitis, myelitis and encephalomyelitis, Inflammation of spinal cord due to toxin, post-immunization encephalitis, post-infectious encephalitis; parent code only: myelitis, inflammatory disease of the central nervous system, encephalitis	IP, ER, OP	Same as base definition	IP
Guillain-Barre syndrome (1)	Guillain-Barre syndrome, Fisher’s syndrome, acute infective polyneuritis	IP, ER, OP	Same as base definition	IP
Transverse myelitis (1)	Transverse myelopathy syndrome	IP, ER, OP	Same as base definition	IP
Pulmonary embolism (1)	Saddle embolus of pulmonary artery, pulmonary infarction, pulmonary embolism; excluding including descendants: venous thrombosis, thrombosis of retinal vein, thrombosed hemorrhoids, septic thrombophlebitis, postpartum deep phlebothrombosis, portal vein thrombosis, phlebitis and thrombophlebitis of intracranial sinuses, obstetric pyemic and septic pulmonary embolism, obstetric pulmonary embolism, obstetric blood-clot pulmonary embolism, obstetric air pulmonary embolism, cerebral venous thrombosis in pregnancy, Budd-Chiari syndrome, antepartum deep vein thrombosis, amniotic fluid embolism	IP, ER, OP	Septic pulmonary embolism with acute cor pulmonale; septic pulmonary embolism without acute cor pulmonale; chronic pulmonary embolism	IP, ER, OP

Abbreviations: IP = inpatient; ER = emergency room; OP = outpatient; IP/ER = inpatient and or ER (used when a place of service cannot separate place of service of emergency room and inpatient encounters).

**Table 2.** Outcomes evaluated by standardization.

Source code comparison				
Outcomes (number of comparisons)	Base definition (SNOMED-CT terms and its descendants unless otherwise noted)		Source code data (Source codes that are not included in base definition)	
	Definition	Place of service	Definition	Place of service
Appendicitis (2)	Appendicitis	IP, IP/ER	Other appendicitis	IP, IP/ER
Anaphylaxis (2)	Anaphylaxis, anaphylactic shock due to serum	IP, IP/ER	Anaphylactic reaction due to other serum, initial encounter; anaphylactic reaction due to vaccination, subsequent encounter; anaphylactic shock, unspecified, subsequent encounter; anaphylactic reaction due to other serum, subsequent encounter	IP, IP/ER
Non-hemorrhagic stroke (2)	Cerebral infarction; excluding including descendants: lacunar infarction, infarct of cerebrum due to iatrogenic cerebrovascular accident	IP	Persistent migraine aura with cerebral infarction, not intractable, with status migrainosus; persistent migraine aura with cerebral infarction, not intractable, without status migrainosus; persistent migraine aura with cerebral infarction, intractable, with status migrainosus; persistent migraine aura with cerebral infarction, intractable, without status migrai; neonatal cerebral infarction, unspecified sidenosus; neonatal cerebral infarction, right side of brain; neonatal cerebral infarction, left side of brain; neonatal cerebral infarction, bilateral	IP
Pulmonary embolism (1)	Saddle embolus of pulmonary artery, pulmonary infarction, pulmonary embolism; excluding including descendants: venous thrombosis, thrombosis of retinal vein, thrombosed hemorrhoids, septic thrombophlebitis, postpartum deep phlebothrombosis, portal vein thrombosis, phlebitis and thrombophlebitis of intracranial sinuses, obstetric pyemic and septic pulmonary embolism, obstetric pulmonary embolism, obstetric blood-clot pulmonary embolism, obstetric air pulmonary embolism, cerebral venous thrombosis in pregnancy, Budd-Chiari syndrome, antepartum deep vein thrombosis, amniotic fluid embolism	IP, ER, OP	Septic pulmonary embolism with acute cor pulmonale; septic pulmonary embolism without acute cor pulmonale; chronic pulmonary embolism	IP, ER, OP

Abbreviations: IP = inpatient; ER = emergency room; OP = outpatient; IP/ER = inpatient and or ER (used when a place of service cannot separate place of service of emergency room and inpatient encounters).

**Table 3.** Outcomes evaluated by code set change.

Code set comparison				
Outcomes (number of comparisons)	Base definition (SNOMED-CT terms and its descendants unless otherwise noted)		Concept set change (Addition standard codes added to base definition)	
	Definition	Place of service	Definition	Place of service
Acute myocardial infarction (2)	Myocardial infarction, ventricular aneurysm due to and following acute myocardial infarction; thrombosis of atrium, auricular appendage, and ventricle due to and following acute myocardial infarction; pulmonary embolism due to and following acute myocardial infarction; mitral valve regurgitation due to and following acute myocardial infarction; hemopericardium due to and following acute myocardial infarction; cardiac rupture due to and following acute myocardial infarction; atrial septal defect due to and following acute myocardial infarction; arrhythmia due to and following acute myocardial infarction; excluding including descendants: old myocardial infarction	IP	Complication codes due to acute myocardial infarctions	IP
Encephalomyelitis (2)	Encephalomyelitis, encephalitis, myelitis and encephalomyelitis; inflammation of spinal cord due to toxin; post-immunization encephalitis, post-infectious encephalitis; parent code only: myelitis, inflammatory disease of the central nervous system, encephalitis	IP	Postimmunization, postinfectious encephalopathy, other myelitis, myelitis unspecified	IP
Immune thrombo-cytopenia (ITP) (1)	Thrombocytopenic purpura, immune thrombocytopenia	IP, ER, OP	Evans syndrome, congenital and hereditary, post-transfusion, and heparin induced	IP, ER, OP
Hemorrhagic stroke (1)	Subarachnoid hemorrhage, subacute non-traumatic intracranial subdural hemorrhage; spontaneous subarachnoid hemorrhage; spontaneous cerebral hemorrhage; non-traumatic subdural hemorrhage; non-traumatic intracerebral ventricular hemorrhage; intracranial hemorrhage; acute nontraumatic subdural hemorrhage	IP	Nontraumatic subarachnoid hemorrhage in various regions; other nontraumatic subarachnoid hemorrhage and nontraumatic subarachnoid hemorrhage, unspecified	IP
Myocarditis/Pericarditis (1)	Viral pericarditis, systemic lupus erythematosus with pericarditis, pericarditis, myocarditis, histoplasmosis with pericarditis, chest pain due to pericarditis	IP, ER, OP	Myocarditis and pericarditis due to the following: meningococcal, scarlet fever, mumps, toxoplasma, sarcoid, rheumatic, chronic adhesive, influenza, lupus, and post cardiotomy syndrome	IP, ER, OP
Non-hemorrhagic stroke (2)	Cerebral infarction; excluding including descendants: lacunar infarction, infarct of cerebrum due to iatrogenic cerebrovascular accident	IP	Cerebral embolism and thrombosis, sequela of cerebrovascular accident, lacunar infarction	IP

Abbreviations: IP = inpatient; ER = emergency room; OP = outpatient; IP/ER = inpatient and or ER (used when a place of service cannot separate place of service of emergency room and inpatient encounters).

To assess the impact of using source codes in capturing events, source code-based definitions (derived by using ICD10-CM codes as per the BEST [Biologics Effectiveness and Safety System] protocol^3^) were considered for the following outcomes: anaphylaxis, appendicitis, DVT, DIC, encephalomyelitis, GBS, and transverse myelitis as these outcomes had definitions that mapped to additional ICD10-CM codes including those listed in the BEST protocol. For example, 2 different definitions were derived for DVT, one using a standard approach that includes additional codes not defined specifically in the BEST protocol and another definition with only those specified in protocol. The objective of the BEST protocol was to actively monitor the rates of AESIs following vaccination in large administrative databases. The protocol defines each outcome for use in databases that is used for active surveillance.

To assess the impact of using a different code set, definitions that included extra (expanded) standard SNOMED-CT codes was considered for MI, encephalomyelitis, hemorrhagic stroke, immune thrombocytopenia, myocarditis/pericarditis, and non-hemorrhagic stroke. The use of standardized vocabulary showed additional codes that may be relevant to each outcome such as sequela codes, or the outcome due to a cause as described by the code (ie, myocarditis due to toxoplasms). The exact codes added for each outcome are included in Table 3. A detailed description of the phenotype definitions utilized in the study can be found in Appendix Table S2. All definitions were developed in the OHDSI tool ATLAS using the appropriate vocabularies to define a concept set.^17^ The choice of what alternative definitions to consider for each outcome was based on proposed definitions by regulatory agencies, prior literature, or utilizing SNOMED-CT hierarchical structure.

Phenotype definitions were implemented and assessed in each data source using an R shiny application within the CohortDiagnostics package.^13^^,^^18^ CohortDiagnostics is a tool that computes and illustrates descriptive statistics about the patients in a database that meet the phenotype definition. The full cohort diagnostics results are located here: https://data.ohdsi.org/Covid19VaccineAesiDiagnostics/. The tool allows for exploring and contextualizing differences in patients’ compositions and characteristics that result from using different phenotype definitions.^13^^,^^17^^,^^18^

### Background incidence rates

We defined the time at risk as a 365-day period after the index date. Each person in the study population contributed time at risk from January 1 to December 31 for each qualifying year in 2017 to 2019. Participants contributed time at risk until the earliest following occurs: an event occurred during that event’s clean window (exclusion time prior to event to ensure a new event which varied by outcome), at death (if available in the data source), or at the end of their observation period in the data source. Appendix Table S3 provides the characteristics of the patient population by database.^6^ A participant could contribute more than one event in each outcome. A person could have multiple events within the time at risk if they satisfy the washout requirement within each phenotype. Incidence rates were estimated as the total number of events divided by the person time at risk per 100 000 person years for each phenotype definition. Incidence rates were reported by data source as a point estimate overall and stratified by age and gender for each outcome definition.

### Rate ratio across definitions

To assess the effect of phenotype modification on incidence rate, we calculated the rate ratio (RR), by dividing the incidence rate using the base definition by the incidence rate using the modified definition (inpatient, source code, extra code set). RRs were calculated for overall rates and stratified by age and gender.

### Sensitivity analysis: rate comparison and baseline characteristics: GBS

The GBS definition had 3 variations to produce the outcome, the differences being how place of service was being utilized. Site specific distributions amongst phenotypes can yield differences in rates and in phenotypic characteristics among these populations. To determine the effect of this variability, GBS was chosen as an example. The crude incidence rates for the 3 derivations of the phenotype of GBS are presented to assess the change between baseline, inpatient, and inpatient in the primary position for the overall incidence rate. The variation of inpatient primary is included as the definition in the BEST protocol. The baseline covariates available in the data occurring up to 365 days prior to index date are compared to each other for each definition pair (base definition and inpatient and inpatient and inpatient in the primary position) by the absolute standardized difference. Covariates are any SNOMED-CT that occur in any domain in the data (condition, procedure, measurement, demographics). Domain assignment is based on the data type being mapped, for example, diagnosis codes are mapped to the condition domain, LOINC codes to measurements and a full description of domain designation can be found here: https://www.ohdsi.org/web/wiki/doku.php?id=documentation:vocabulary:domains. The number of covariates with a mean >0 and a standardized difference >0.1 are summarized to show the differences amongst the comparison of the 2 populations being compared.

## Results

The analysis is presented on a total of 13 phenotypes in the 3 types of modifications (narcolepsy and facial nerve palsy are omitted from the original set of outcomes listed in the BEST protocol due to lack of appropriate comparisons in this study). Each database contributes to each subtype analysis based on availability of the data. The sensitivity analysis is only conducted on the GBS phenotype in 4 US datasets due to the availability of all places of services.

The 9 databases that do not use ICD-10-CM vocabulary (IPCI_NETHERLANDS, JMDC_JAPAN, BIOBANK_UK, IQVIA_GERMANY, IQVIA_AUSTRIALIA, CPRD_UK, APHM_FRANCE, CC_SERBIA, and HIC_ SCOTLAND) did not show counts for any definitions based on ICD10-CM source code. Additionally, inpatient data were not available in 4 data sources (CPRD_UK, IQVIA_GERMANY, IQVIA_AUSTRIALIA, and IPCI_NETHERLANDS), resulting in zero counts for inpatient based definitions.

### Inpatient restriction

**Figure 1.**
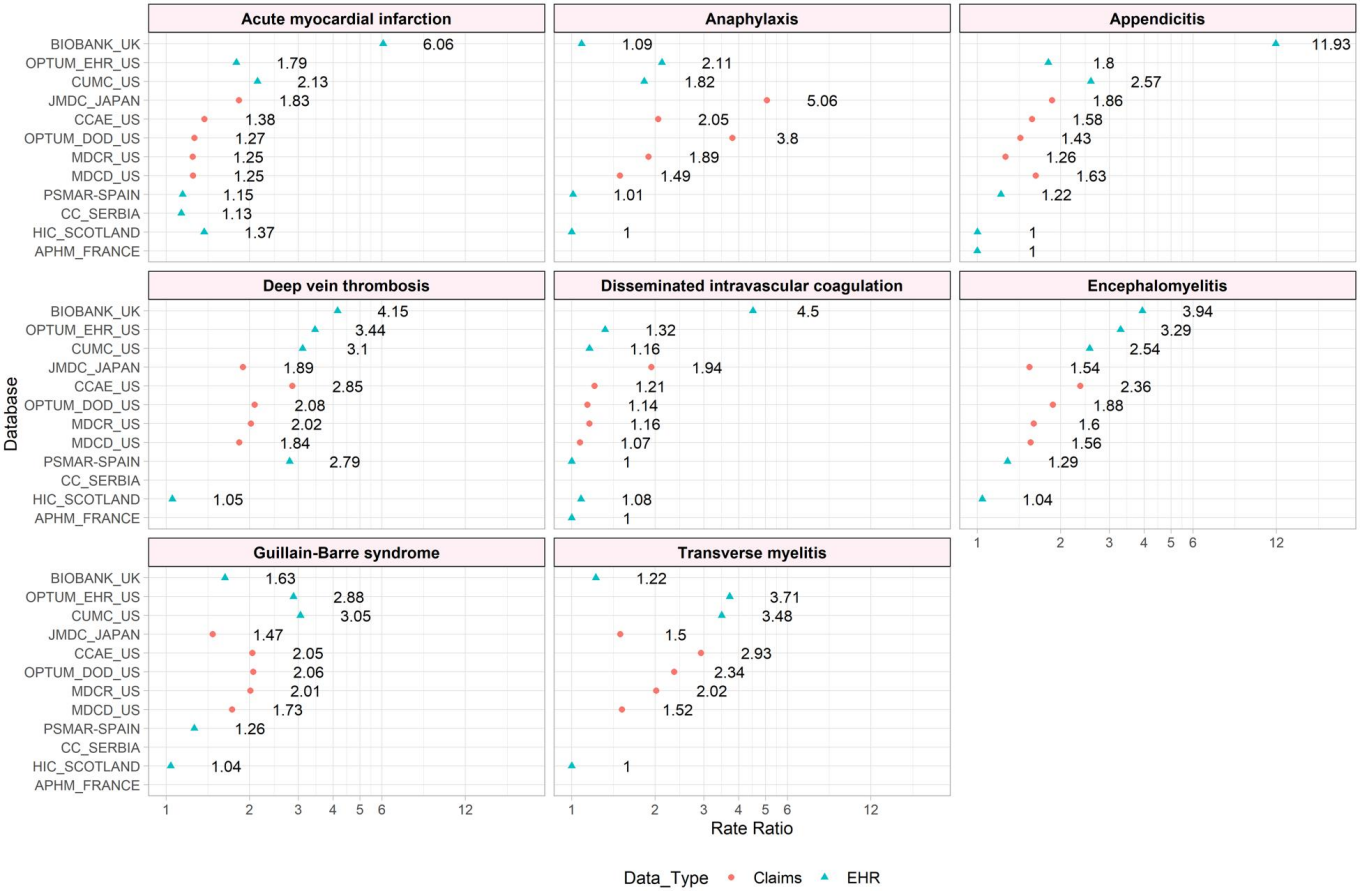
Incidence rate ratios for overall incidence rates per 100 000 person-years by phenotype restricted by inpatient versus base definition for claims and EHR databases.

Figure 1 illustrates the overall RR when comparing incidence rates (IR) using inpatient-based definition to IR using the base definition for each outcome by database for the overall rate not stratified by age and gender. The RR varies by outcome and by data sources and ranges from 1 to 11.93. The range of RR varies by outcome with the highest (1-11.93) in appendicitis, and the lowest in GBS (1.05-3.05). Claims databases show less variance than electronic health records (EHRs).

### Standardization

**Figure 2.**
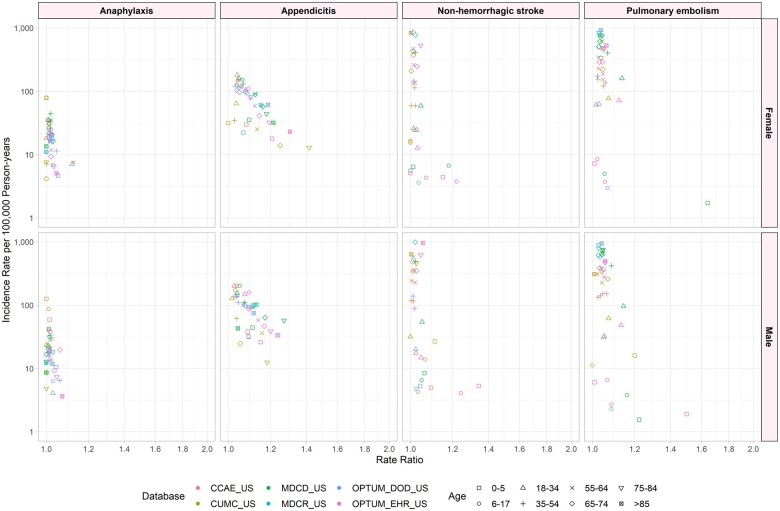
Incidence rate ratios for incidence rates per 100 000 person-years by phenotype and database for source code restriction versus base definition, stratified by age and sex.

Figure 2 illustrates the age-gender-specific RRs when comparing IRs using source code-based definitions to the base definitions for each outcome by database. The highest RR was in pulmonary embolism at 1.64 times higher than the standard definition in ages 0-5 across all databases and phenotypes evaluated. Anaphylaxis had the smallest change in incidence rates with the highest RR at 1.12 and showed the lowest variability by age/gender/database. Appendicitis showed incidence RRs from 1 to 1.41 across all ages and databases with highest RR in older ages (55+) and the highest variability by age/gender/database. Non-hemorrhagic stroke had low variability with the highest RR at 1.34, but most RRs were near 1 across age/gender/database.

### Code set change

**Figure 3.**
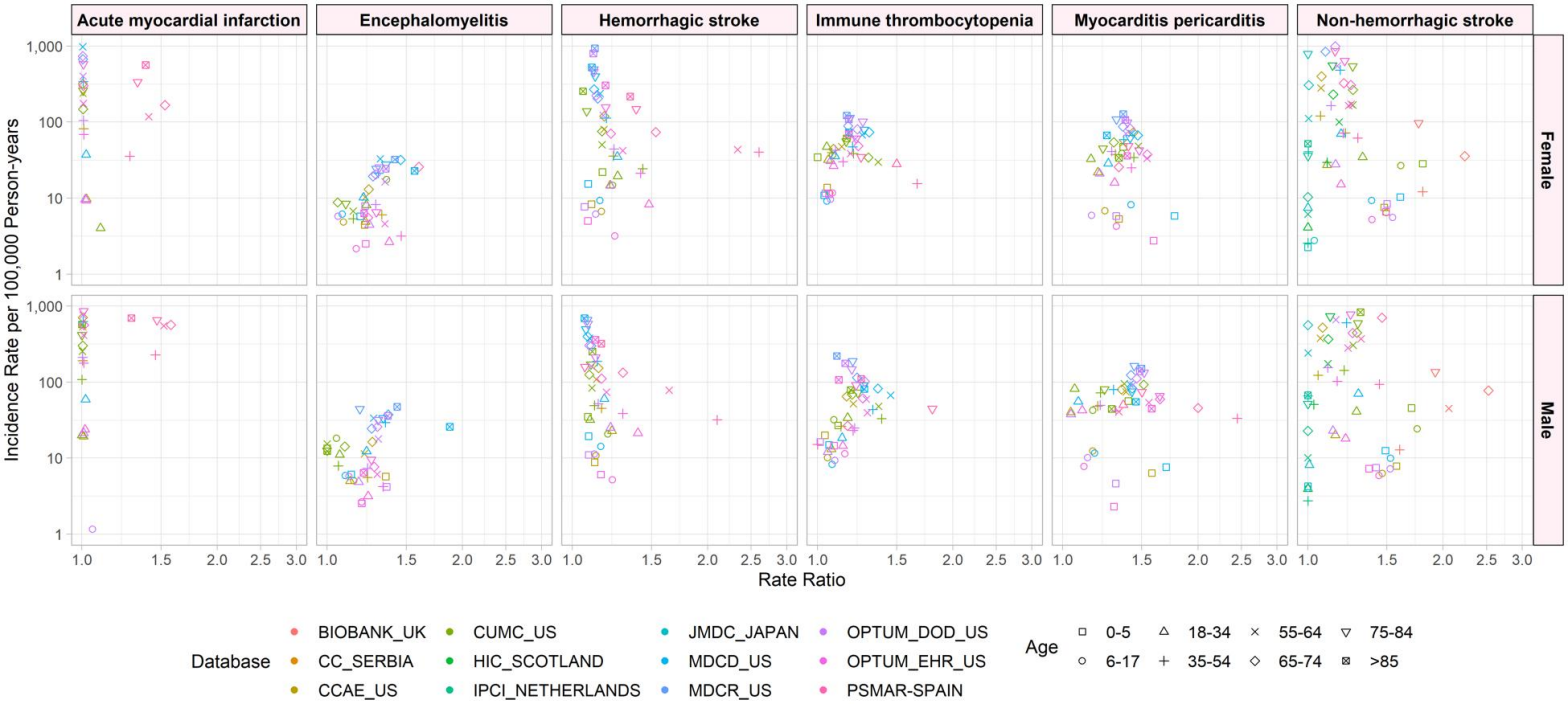
Incidence rate ratios for overall incidence rates per 100 000 person-years by phenotype and database for code set change versus base definition, stratified by age and sex.

Figure 3 illustrates the age-gender-specific RRs when comparing IRs using an expanded code-set definition to the base definition for each outcome by database. Changing the code set resulted in additional codes being added to the base definition. The highest RR was in hemorrhagic stroke at 2.73, higher in males than females, followed by myocarditis at 2.24. All phenotypes except for anaphylaxis showed large heterogeneity by age and gender. Ages 35+ across both genders showed the highest variability for these outcomes.

### Sensitivity analysis: rate comparison and baseline characteristics: GBS

The incidence rates for the 3 modifications (base definition, inpatient, and inpatient admission in the primary position) varied both by databases and modification type in Table 4. The highest variation occurred in MDCR_US with a range of incidence rates from 3.74 to 28.14 and the lowest in MDCD_US from 2.57 to 7.40. The maximum standardized difference ranged from 0.31 to 0.65 when comparing inpatient to overall, while the range for inpatient compared to inpatient primary was 0.14-0.27. Each restriction added at least a 2-fold increase in the incidence rate by applied each subsequent restriction.

**Table 4.** Incidence rates per 100 000 person years for Guillain-Barré syndrome and baseline characteristics.

Guillain-Barré syndrome	Databases				
	CCAE_US	MDCD_US	MDCR_US	Optum_DOD_US	Optum_EHR_US
Incidence rates (per 100 000 person years)					
All places of service (base definition)	7.14	7.40	28.14	14.34	7.37
Inpatient only	3.48	4.28	13.99	6.96	2.53
Inpatient in the primary position	1.81	2.57	3.74	3.62	1.12
Baseline characteristics comparisons					
All vs IP					
Number of features (mean > 0; covariates occurring in both definitions)	18 246	16 448	14 596	34 409	21 020
Features with absStDiff > 0.1 (*n*)	462	281	320	650	2241
Maximum (absStDiff)	0.54	0.31	0.39	0.65	0.51
IP vs IP primary					
Number of features (mean > 0; covariates occurring in both definitions)	15 932	10 628	8891	25 095	14 634
Features with absStDiff > 0.1 (*n*)	32	203	135	47	340
Maximum (absStDiff)	0.14	0.27	0.27	0.19	0.20

## Discussion

We utilized real-world data to evaluate the effect of phenotype modification on the background rate of 13 outcomes, with multiple plausible definitions across a large collection of data sources. We compared the background incidence rates to examine the impact of place of care, standardization, and code sets on heterogeneity in estimated incidence. Our results suggest that some modifications to phenotype definition can lead to significant changes in incidence rate estimates. This highlights the importance of determining accurate phenotype definitions in safety research.

The OHDSI network allows for rapid, transparent, and reproducible analyses over a large network of data sources. This study demonstrates how a research network can be used to empirically evaluate alternative outcome definitions quickly and in a standard manner, which is achieved using standard vocabularies. Phenotype definitions should be evaluated for the impact of changes that are not readily apparent within a single database (eg, code sets). Tools such as CohortDiagnostics enable investigators to explore phenotypes that result from implementing alternative definitions for the same clinical idea. Evaluating definitions over a network of data sources can highlight issues, such as the fact that not all data types are available in all data sources, which can lead to a different composition of patients amongst definitions.

Often, researchers make choices to restrict or not to restrict to inpatient events in an attempt to reduce measurement errors, usually by increasing specificity. This study highlights the need to thoroughly evaluate the implication of such a choice. The effect of an inpatient restriction showed the highest amount of variation among databases and phenotypes, suggesting some level of error. For example, anaphylaxis shows higher incidence rate in claims databases than EMR databases and overall, the incidence RRs range from 1.00 to 5.04. This analysis alone cannot determine the total error by restricting to inpatient, but it likely increases specificity and decreases sensitivity, but we do not know by how much for each. Also, coding errors and how data get recorded in all databases (ie, claims are generally processed for payment) also are a form of error for outcomes represented here. Outcomes that are expected to occur in an inpatient setting such as appendicitis can show up to a 2-fold increase in IR when not restricting to events occurring in hospitals that record inpatient data. Outpatient EHRs may record a presence of a procedure, such as appendectomy but the actual diagnosis happens prior to the record of procedure, which could influence the capture of events. Rarer events tended to have the least amount of variation, as seen by DIC or encephalomyelitis. This is likely due to diagnostic and data capture; the definitions of these outcomes are acute and require substantial diagnostic workup. The variability by country was lower than expected as databases in this study vary by country likely due to coding practices and data types. A small number of events in certain strata can result in high variability of rates as seen with pulmonary embolism in the 0-5 age range. This variability is invisible unless definitions are exposed to systematic evaluation as shown in this study.

Standardization of data compared to using source data showed little to no difference in incidence estimates. For example, pulmonary embolism rates for both males and females range from 1.0 to 1.2 across all databases and are the same for anaphylaxis. Standardization can help facilitate analysis across a network of databases. The underlying populations of each database may be different, but the clinical concepts for conditions like appendicitis and acute myocardial infarction are expected to be similar around the world. However, analyses stratified by age and sex suggest that database heterogeneity can go beyond differences in underlying populations to produce differences in phenotype incidence. Code set variation also had high variability by outcomes and across databases. The rates for hemorrhagic stroke showed the highest range from 1.0 to 3.0 across age, sex, and database. While each outcome has a different underlying true incidence rate, which may contribute to differences in degree of heterogeneity across outcomes, variability across outcomes may also be due to variability in the use of different code sets for different outcomes across regions or practices.

The sensitivity analysis of GBS showed wide variability across modifications, with an incidence twice as high when using inpatient primary compared to any inpatient and 4 times as high when using any place of service compared to inpatient primary only across all databases. The CDC protocol defined GBS using inpatient primary, while the BEST protocol used inpatient, and these 2 variations lead to a notable effect on the background rates, at least doubling the rate when transitioning from primary restriction to inpatient only. Characteristics of patients identified via the different definitions showed substantial differences in covariate distributions. Differences in characteristics between the inpatient and inpatient primary were less pronounced and related mostly to drainage of spinal fluid. However, the trade-off of including a primary code which is only available in selected databases in the United States is a challenge and one that should be considered prior to implementing a phenotype. The overall performance of a phenotype definition could show an increase in sensitivity with this restriction but is a compromise when utilizing other databases that do not have these specific markers. Utilizing other methods to determine the performance characteristics such as background covariates of a phenotype could be beneficial prior to selecting a phenotype definition. These choices should be evaluated prior to deciding and are likely influenced by what the outcome definition is.

The strengths of the study include conducting the analysis on a global network of data sources, and the ability to rapidly assess a large set of definitions for multiple outcomes in a systematic manner. The limitations of the study include that standard vocabularies could change over time and definitions should be revisited to ensure mappings represent the population they intend to study. Also, while this study examined changes in outcome incidence with alternative definitions, we did not examine the performance characteristics (eg, sensitivity and specificity) of each phenotype. When possible, such performance characteristics should be used to select an optimal phenotype. The stratification of place of service is an aggregation of various types of care (office visits, specialist visits, diagnostics done in an emergency room) and while this level of granularity would be beneficial to evaluate most of our databases do not contain this level of information. The study demonstrates the need for researchers to consider broad changes on a phenotype definition. Variability by databases, coding choices and standardization can affect the resulting IR analysis. The 3rd modification choice illustrates how a given phenotype definition can vary the resulting analysis such as in the example of inpatient modification which results in a large variation of IR compared to very little when standardization is applied. Further resources should be devoted to exploring the impact of the definition prior to conducting a study.

Comparing phenotype definitions is critical for understating the trade-offs researchers make when evaluating an outcome definition, and the ability to process this information and understand the strengths and weaknesses of a definition over a network of data sources is important to be able to implement the strongest definition in each data source for a study or use in safety surveillance.

## Conclusion

Our study systematically compared incidence rates among different phenotype definitions. We found variation when introducing restrictions based on the setting of events for selected phenotypes. There is considerable database-level heterogeneity within a phenotype when changing the concept set on the incidence rates, providing an additional layer of validity and comparability across databases prior to conducting estimation studies.

## Supplementary Material

ooad096_Supplementary_Data

## Data Availability

The data that support the findings of this study are available to license from IBM, Optum, and CPRD. Data are available from IBM at https://www.ibm.com/products/marketscan-research-databases, from Optum at https://www.optum.com/business/solutions/life-sciences/real-world-data.html, and CPRD at https://www.cprd.com/. The New England Institutional Review Board determined that studies conducted in MDCR, MDCD, and OPTUM_DOD were exempt from study-specific Institutional Review Board review, as these studies do not qualify as human subject research. Likewise, the CPRD was not considered by the New England Institutional Review Board because the CPRD has its own process. This study utilized data from the Clinical Practice Research Datalink (CPRD) obtained under license from the UK Medicines and Healthcare Products Regulatory Agency. The use of Clinical Practice Research Datalink—AURUM was approved by the Research Data Governance Process, protocol 20_000211 was approved by the Independent Scientific Advisory Committee. However, the interpretation and conclusions contained in this study are those of the authors alone.
