# Supplementary material for: Evaluating the impact of alternative phenotype definitions on incidence rates across a global data network
Source: JAMIA Open. 2023 Nov 21;6(4):ooad096. doi: 10.1093/jamiaopen/ooad096 (PMC10662662; doi:10.1093/jamiaopen/ooad096)
Supplement: ooad096_Supplementary_Data [file ooad096_supplementary_data.docx]

## Appendix

## Appendix Table 1. Database descriptions

| **Name** | **Description** |
| --- | --- |
| Health Data Warehouse of Assistance Publique - Hopitaux de Marseille (APHM_FRANCE) | The Assistance Publique – Hôpitaux de Marseille (AP-HM) is a public university hospital system with 4 hospitals, 3,400 beds, and more than 12,000 health care professionals. The AP-HM is one the largest health centers in France (after Paris and Lyon). For adults and children, the AP-HM provide hospital care services going from primary care to cutting-edge treatments of complex and rare pathologies. Approximately 300,000 hospitalizations are recorded every year at the AP-HM, involving approximately 210,000 patients. Our information system includes multiple data sources with electronic medical record (Axigate), treatment prescription and deliverance (Pharma), oncology treatment (Chimio), Biology, PMSI (Programme de Médicalisation des Systèmes d’Information). The PMSI is the French medico-administrative database for all hospitalizations based on diagnosis related-groups (DRG) that we can group into significant diagnostic categories. All the stays are coded using the International Classification of Disease (ICD-10th version). All these data are collected and stored for more than 10 years with more than 1 billion pieces of data. |
| Clinical Practice Research Datalink - AURUM (CPRD_UK) | The Clinical Practice Research Datalink (CPRD) is a governmental, not-for-profit research service, jointly funded by the National Health Service (NHS) National Institute for Health Research (NIHR) and the Medicines and Healthcare products Regulatory Agency (MHRA), a part of the Department of Health, United Kingdom (UK). CPRD Aurum consists of data collected from UK primary care offices using EMIS® software. This includes conditions, observations, measurements, and procedures that the general practitioner is made aware of in addition to any prescriptions as prescribed by the general practitioner. |
| Columbia University Irving Medical Center (CUMC_US) | The Columbia University Irving Medical Center (CUIMC) database comprises electronic health records on 6,666,613 patients, with data collection starting in 1985. CUIMC is a northeast United States of America (US) quaternary care center with primary care practices in northern Manhattan and surrounding areas, and the database includes inpatient and outpatient care. The database currently holds information about the person (demographics), visits (inpatient and outpatient), conditions (billing diagnoses and problem lists), drugs (outpatient prescriptions and inpatient orders and administrations), devices, measurements (laboratory tests and vital signs), and other observations (symptoms). The data sources include current and previous electronic health record systems (homegrown Clinical Information System, homegrown WebCIS, Allscripts Sunrise Clinical Manager, Allscripts TouchWorks, Epic Systems), administrative systems (IBM PCS-ADS, Eagle Registration, IDX Systems, Epic Systems), and ancillary systems (homegrown LIS, Sunquest, Cerner Laboratory). The data were extracted from each system and transformed to the Observational Health Data Science and Informatics (OHDSI) Observational Medical Outcomes Partnership (OMOP) Common Data Model (CDM): common data model source name Epic Legacy CUMC MERGE common data model ETL reference v1.3.0.cdm5.3 common data model release date 2020-05-22 vocabulary version v5.0 30-APR-20 with OMOP common data model version 5.3.1 and local version name ohdsi_cumc_2020q4r1. |
| Health Informatics Centre (HIC_SCOTLAND) | Health datasets from the Tayside and Fife regions of Scotland, provided by the Health Informatics Centre (HIC) at the University of Dundee. |
| IBM® MarketScan® Commercial Claims and Encounters Database (IBM_CCAE) | The IBM® MarketScan® Commercial Database (CCAE) includes health insurance claims across the continuum of care (i.e., inpatient, outpatient, outpatient pharmacy, carve-out behavioral healthcare) as well as enrollment data from large employers and health plans across the United States who provide private healthcare coverage for more than 155 million employees, their spouses, and dependents. This administrative claims database includes a variety of fee- for-service, preferred provider organizations, and capitated health plans. |
| IBM® MarketScan® Multi-State Medicaid Database (IBM_MDCD) | The IBM® MarketScan® Multi-State Medicaid Database (MDCD) reflects the healthcare service use of individuals covered by Medicaid programs in numerous geographically dispersed states. The database contains the pooled healthcare experience of Medicaid enrollees, covered under fee-for-service and managed care plans. It includes records of inpatient services, inpatient admissions, outpatient services, and prescription drug claims, as well as information on long-term care. Data on eligibility and service and provider type are also included. In addition to standard demographic variables such as age and gender, the database includes variables such as federal aid category (income based, disability, Temporary Assistance for Needy Families) and race. |
| IBM® MarketScan® Medicare Supplemental and Coordination of Benefits Database (IBM_MDCR) | The IBM® MarketScan® Medicare Supplemental Database (MDCR) represents the health services of approximately 10 million retirees in the United States with Medicare supplemental coverage through employer-sponsored plans. This database contains primarily fee-for-service plans and includes health insurance claims across the continuum of care (e.g. inpatient, outpatient and outpatient pharmacy). |
| Integrated Primary Care Information (IPCI_NETHERLANDS) | The Integrated Primary Care Information (IPCI) database started in 1992 and is collected from EHR records of patients registered with their general practitioners (GPs) throughout the Netherlands. The selection of 640 practices, of which 422 are currently still actively contributing, is representative for the entire country. The database contains records from in total 2.5 million patients of which approximately 1.4 million are still active (July 2021) out of a Dutch population of 17 million.  The observation period for a patient is determined by the date of registration at the GP and the date of leave/death. The observation period start date is refined by many quality indicators, e.g., exclusion of peaks of conditions when registering at the GP. All data before the observation period is kept as history data. Drugs are captured as prescription records with product, quantity, dosing directions, strength, and indication. |
| IQVIA(R) Australia Longitudinal Patient Data (LPD) (IQVIA_AUSTRAILIA) | The IQVIA(R) Australia Longitudinal Patient Data (LPD) database consists of data collected from Australian general practitioner (GP) offices for all ages. Data include prescriptions as prescribed, conditions, observations, measurements by the GP, in the outpatient setting. No specialist or hospital information is included. |
| IQVIA® Disease Analyzer (DA) Germany (IQVIA_GERMANY) | The IQVIA® Disease Analyzer (DA) Germany database consists of data collected from physician practices and medical centers for all ages. Mostly primary care physician data however some data from specialty practices (where practices are electronically connected to each other) and some lab data is included. Key attributes include demographics, prescriptions as prescribed at brand level, diagnosis, lab measurements, and actions (e.g. referrals, sick notes). |
| Japan Medical Data Center (JMDC_JAPAN) | Japan Medical Data Center (JMDC) database consists of data from 60 Society-Managed Health Insurances covering workers aged 18 to 65 and their dependents (children younger than 18 years old and elderly people older than 65 years old). Those aged 66 or older are less representative as compared with whole population in the nation. When estimated among the people who are younger than 66 years old, the proportion of children younger than 18 years old in JMDC is approximately the same as the proportion in the whole nation. JMDC data includes data on membership status of the insured people and claims data provided by insurers under contract. Claims data are derived from monthly claims issued by clinics, hospitals and community pharmacies. The number of claims issued and added to JMDC database is about 800,000 per month. The size of JMDC population is 1.9 million, 1.5% of about 120 million people in the whole nation. |
| Optum® de-identified Electronic Health Record Dataset (OPTUM_EHR_US) | Optum's longitudinal electronic health record (EHR) repository is derived from dozens of healthcare provider organizations in the United States (US), that include more than 700 hospitals and 7,000 clinics: treating more than 102 million patients receiving care in the US. The data is certified as de-identified by an independent statistical expert following the Health Insurance Portability and Accountability Act (HIPAA) statistical de-identification rules and managed according to Optum® customer data use agreements. Clinical, claims, and other medical administrative data is obtained from both Inpatient and Ambulatory EHRs, practice management systems and numerous other internal systems. Information is processed, normalized, and standardized across the continuum of care from both acute inpatient stays and outpatient visits. Optum® data elements include demographics, medications prescribed and administered, immunizations, allergies, lab results (including microbiology), vital signs and other observable measurements, clinical and inpatient stay administrative data, and coded diagnoses and procedures. In addition, Optum® uses natural language processing (NLP) computing technology to transform critical facts from physician notes into usable datasets. The NLP data provides detailed information regarding signs and symptoms, family history, disease related scores (i.e. RAPID3 for RA, or CHADS2 for stroke risk), genetic testing, medication changes, and physician rationale behind prescribing decisions that might never be recorded in the EHR. |
| Optum De-Identified Clinformatics® Data Mart Database - Date of Death (OPTUM_DOD_US) | Optum's Clinformatics® Data Mart is derived from a database of administrative health claims for members of large commercial and Medicare Advantage health plans. The database includes approximately 17-19 million annual covered lives, for a total of over 65 million unique lives over a 12-year period (1/2007 through 12/2019). Clinformatics® Data Mart is statistically de-identified under the Expert Determination method consistent with the Health Insurance Portability and Accountability Act (HIPAA) and managed according to Optum customer data use agreements. Administrative claims submitted for payment by providers and pharmacies are verified, adjudicated and de-identified prior to inclusion. This data, including patient-level enrollment information, is derived from claims submitted for all medical and pharmacy health care services with information related to healthcare costs and resource utilization. The population is geographically diverse, spanning all 50 states. Optum Clinformatics® Data Mart Socio-Economic Status provides socio-economic status for members with both medical and pharmacy coverage and location information for patients at the United States of America Census Division level. |
| UK Biobank (BIOBANK_UK) | The United Kingdom (UK) Biobank (UKB) is a population-level, longitudinal research study of 500,000 participants in age range 40-69 from England, Scotland, and Wales recruited between 2006 - 2010. The study contains detailed baseline phenotypic (physical and biomarker measurements, diet and alcohol, cognitive function, mental health, education and employment), imaging (abdomen, brain and heart MRI, DXA), and genotypic (imputed genome, whole genome, exome sequencing) information. All participants have longitudinal follow-up data through electronic health records containing events from primary (231k patients), secondary care (395k patients), and national death and cancer registration information data. Since 2012, UKB has approved over 13,000 registrations from researchers working in over 1,375 institutes in 68 countries and approved over 1,200 applications to enable these researchers to access the data. Recently, the UKB has made data from national COVID-19 testing on participants (at the moment containing test results from 1,474 participants). During the current COVID-19 pandemic, the UKB is releasing data from national COVID-19 testing, primary care EHR, hospital inpatient episodes, national mortality registers and intensive care data (for participants with confirmed COVID-19) on a monthly basis. |
| University Clinical Center of Serbia (CC_SERBIA) | University Clinical Center of Serbia is the largest health care facility in Serbia and Europe (by number of beds). It covers all fields of medicine in a tertiary setting, using Heliant as HIS/EHR (Health information system / Electronic health record), which follows many hospital business processes, from patient care, financial information, and to drug and material maintenance. |
| Parc de Salut Mar Barcelona Information System (PSMAR_SPAIN) | The IMASIS database is the Electronic Health Record (EHR) system of the Parc Salut Mar Barcelona (PSMAR) which is a complete healthcare services organization including two general hospitals, one mental health care center and one social-healthcare center, which are offering specific and different healthcare services in the Barcelona city area (Spain). IMASIS includes information since 1990 and from different settings such as admissions, outpatients, emergency room, and major ambulatory surgery. Currently, the database contains hospital-based information on approximately 1 million patients. |

## Appendix Table 2. Outcome definition links

| **Phenotype** | **Comparison** | **ID 1*** | **ID 1 Link** | **ID 2*** | **ID 2 Link** |
| --- | --- | --- | --- | --- | --- |
| Hemorrhagic stroke | Base defintion/Code set | 405 | https://atlas.ohdsi.org/#/cohortdefinition/405/definition | 396 | https://atlas.ohdsi.org/#/cohortdefinition/396/definition |
| Immune thrombocytopenia (ITP) | Base defintion/Code set | 335 | https://atlas.ohdsi.org/#/cohortdefinition/335/definition | 401 | https://atlas.ohdsi.org/#/cohortdefinition/401/definition |
| Myocarditis/Pericarditis | Base defintion/Code set | 339 | https://atlas.ohdsi.org/#/cohortdefinition/339/definition | 399 | https://atlas.ohdsi.org/#/cohortdefinition/399/definition |
| Non-hemorrhagic stroke | Base defintion/Code set | 406 | https://atlas.ohdsi.org/#/cohortdefinition/406/definition | 342 | https://atlas.ohdsi.org/#/cohortdefinition/342/definition |
| Acute myocardial infarction | Base defintion/Code set | 340 | https://atlas.ohdsi.org/#/cohortdefinition/340/definition | 388 | https://atlas.ohdsi.org/#/cohortdefinition/388/definition |
| Encephalomyelitis | Base defintion/Code set | 346 | https://atlas.ohdsi.org/#/cohortdefinition/346/definition | 393 | https://atlas.ohdsi.org/#/cohortdefinition/393/definition |
| Acute myocardial infarction | Base definition/Inpatient | 383 | https://atlas.ohdsi.org/#/cohortdefinition/383/definition | 340 | https://atlas.ohdsi.org/#/cohortdefinition/340/definition |
| Anaphylaxis | Base definition/Inpatient | 349 | https://atlas.ohdsi.org/#/cohortdefinition/349/definition | 407 | https://atlas.ohdsi.org/#/cohortdefinition/407/definition |
| Appendicitis | Base definition/Inpatient | 386 | https://atlas.ohdsi.org/#/cohortdefinition/386/definition | 344 | https://atlas.ohdsi.org/#/cohortdefinition/344/definition |
| Deep vein thrombosis | Base definition/Inpatient | 402 | https://atlas.ohdsi.org/#/cohortdefinition/402/definition | 403 | https://atlas.ohdsi.org/#/cohortdefinition/403/definition |
| Encephalomyelitis | Base definition/Inpatient | 382 | https://atlas.ohdsi.org/#/cohortdefinition/382/definition | 346 | https://atlas.ohdsi.org/#/cohortdefinition/346/definition |
| Guillain Barre syndrome | Base definition/Inpatient | 380 | https://atlas.ohdsi.org/#/cohortdefinition/380/definition | 343 | https://atlas.ohdsi.org/#/cohortdefinition/343/definition |
| Transverse myelitis | Base definition/Inpatient | 381 | https://atlas.ohdsi.org/#/cohortdefinition/381/definition | 334 | https://atlas.ohdsi.org/#/cohortdefinition/334/definition |
| Disseminated intravascular coagulation | Base definition/Inpatient | 385 | https://atlas.ohdsi.org/#/cohortdefinition/385/definition | 336 | https://atlas.ohdsi.org/#/cohortdefinition/336/definition |
| Anaphylaxis | Base definition/Source code | 407 | https://atlas.ohdsi.org/#/cohortdefinition/407/definition | 389 | https://atlas.ohdsi.org/#/cohortdefinition/389/definition |
| Appendicitis | Base definition/Source code | 427 | https://atlas.ohdsi.org/#/cohortdefinition/427/definition | 390 | https://atlas.ohdsi.org/#/cohortdefinition/390/definition |
| Non-hemorrhagic stroke | Base definition/Source code | 406 | https://atlas.ohdsi.org/#/cohortdefinition/406/definition | 397 | https://atlas.ohdsi.org/#/cohortdefinition/397/definition |
| Pulmonary embolism | Base definition/Source code | 336 | https://atlas.ohdsi.org/#/cohortdefinition/336/definition | 400 | https://atlas.ohdsi.org/#/cohortdefinition/400/definition |

*ID 1 refers to base cohort and ID 2 refers to comparison cohort. The comparison is described is in the comparison column.

## Appendix Table 3. Characteristics of population, stratified by database and analysis type


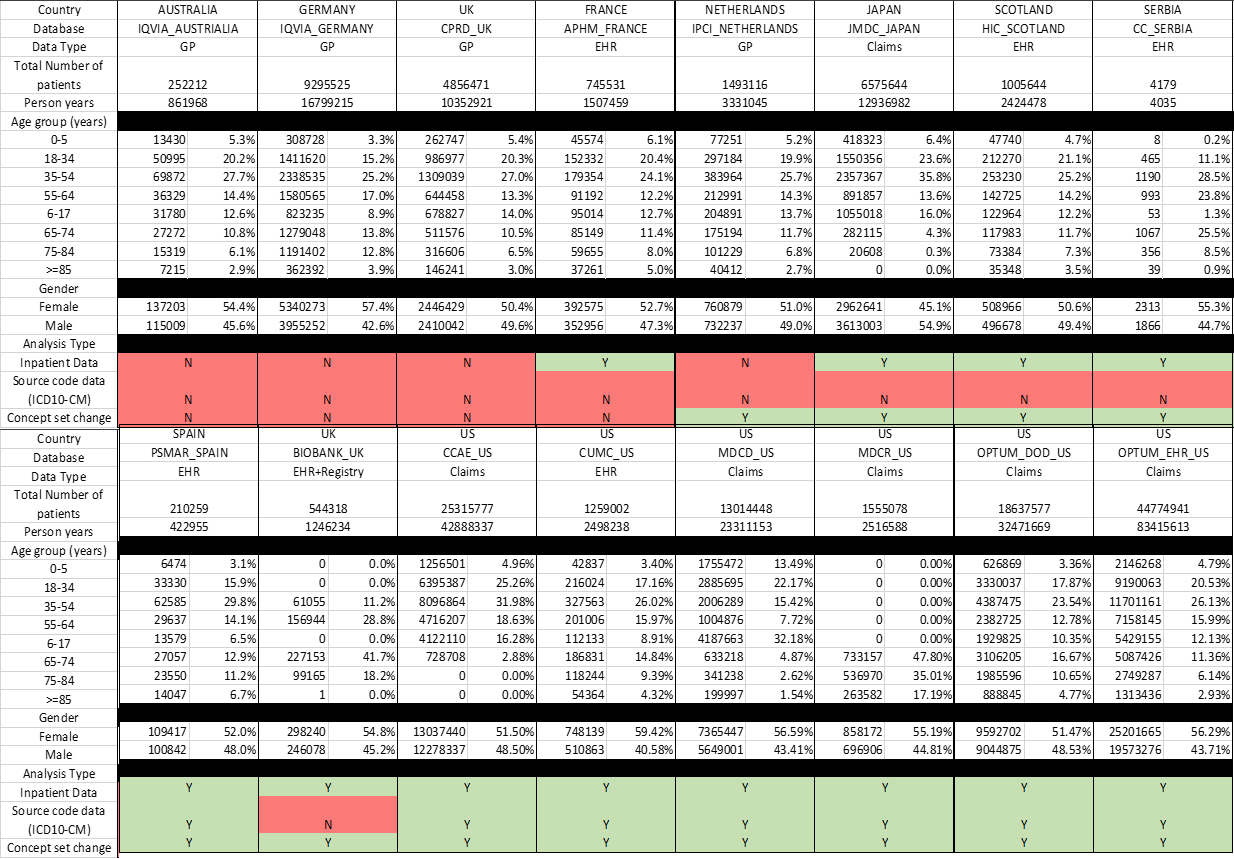


## Appendix Figure 1. Study design for population at risk

**2017**

**2018**

**2019**

**31 Dec 2019**

First cohort entry

Jan 1 2017

365 days time at risk

**X**

**X**

**X**

End of observation

period

Clean window

Clean window

Events

Person time contributed

Observed/enrollment in database
